# The Anti-Inflammatory and Uric Acid Lowering Effects of Si-Miao-San on Gout

**DOI:** 10.3389/fimmu.2021.777522

**Published:** 2022-01-05

**Authors:** Ling Cao, Tianyi Zhao, Yu Xue, Luan Xue, Yueying Chen, Feng Quan, Yu Xiao, Weiguo Wan, Man Han, Quan Jiang, Liwei Lu, Hejian Zou, Xiaoxia Zhu

**Affiliations:** ^1^ Division of Rheumatology, Huashan Hospital, Fudan University, Shanghai, China; ^2^ Institute of Rheumatology, Immunology and Allergy, Fudan University, Shanghai, China; ^3^ Department of Rheumatology, Yueyang Hospital of Integrated Traditional Chinese and Western Medicine, Shanghai University of Traditional Chinese Medicine, Shanghai, China; ^4^ Institute of Spacecraft Equipment, Shanghai, China; ^5^ Guang’anmen Hospital, China Academy of Chinese Medical Sciences, Beijing, China; ^6^ Department of Pathology and Shenzhen Institute of Research and Innovation, The University of Hong Kong, Hong Kong, Hong Kong SAR, China

**Keywords:** gout, hyperuricemia, Si-Miao-San, inflammation, uric acid

## Abstract

**Background:**

Si-Miao-San (SMS) is a well-known traditional Chinese medicine. This study aims to evaluate the anti-inflammatory effects of SMS on gouty arthritis and its potential mechanism of action.

**Methods:**

The effects and mechanism of SMS were evaluated in monosodium urate (MSU)-treated mice or macrophages. The expression of cytokines and PI3K/Akt was analyzed using real-time PCR and Western blotting analyses. Macrophage polarization was assessed with immunofluorescence assays, real-time PCR, and Western blotting. Mass spectrometry was used to screen the active ingredients of SMS.

**Results:**

Pretreatment with SMS ameliorated MSU-induced acute gouty arthritis in mice with increased PI3K/Akt activation and M2 macrophage polarization in the joint tissues. *In vitro*, SMS treatment significantly inhibited MSU-triggered inflammatory response, increased p-Akt and Arg-1 expression in macrophages, and promoted M2 macrophage polarization. These effects of SMS were inhibited when PI3K/Akt activation was blocked by LY294002 in the macrophages. Moreover, SMS significantly reduced serum uric acid levels in the hyperuricemia mice. Using mass spectrometry, the plant hormones ecdysone and estrone were detected as the potentially effective ingredients of SMS.

**Conclusion:**

SMS ameliorated MSU-induced gouty arthritis and inhibited hyperuricemia. The anti-inflammatory mechanism of SMS may exert anti-inflammatory effects by promoting M2 polarization *via* PI3K/Akt signaling. Ecdysone and estrone might be the potentially effective ingredients of SMS. This research may provide evidence for the application of SMS in the treatment of gout.

## Introduction

Gouty arthritis is a common inflammatory arthropathy induced by monosodium urate (MSU) crystals deposited in joints and soft tissues (1–3). Hyperuricemia is the key pathophysiological condition for the development of symptomatic gout (4, 5). The prevalence of hyperuricemia was about 2.6%-36.0% (6), the prevalence of gout was about 0.03%-15.3% (7). Clinical remission can be achieved in most patients through anti-inflammatory treatment and urate-lowering therapy (ULT) (8–10). However, some patients experience recurrent flares during ULT. Currently, the anti-inflammatory treatments, such as colchicine, glucocorticoid, and non-steroidal anti-inflammatory drugs are restricted in the patients with digestive diseases (e.g., peptic ulcer, gastrointestinal bleeding) or renal insufficiency (11). Therefore, it is imperative to explore new approaches for the treatment of gouty arthritis, especially with complementary and alternative medicine.

Si-Miao-San (SMS), a well-known traditional Chinese medicine, was first described in the monograph “Dan Xi Xin Fa” (Chinese comprehensive medicinal book) in the Yuan Dynasty in China (12). SMS has been widely used in the treatment of gout and gouty arthritis for approximately 700 years, showing clinically confirmed efficacy as effective therapy in patients with gout (13–15). SMS comprises four individual herbs, namely *Phellodendron chinese* SCHNEID (Rutaceae), *Atractylodes lancea* (Thunb.) DC. (Asteraceae), *Achyranthes bidentata* BL. (Amaranthaceae), and *Coix lacryma-jobi* L. (Poaceae), each of which was reported to be safe in clinical application in Chinese medicine theory (16). Previous studies have reported that SMS can significantly relieve the symptoms of gouty arthritis through its anti-inflammatory effects (17–19).

Clinically, SMS has been safely used in gouty patients with concomitant complications such as chronic renal insufficiency, heart problems, gastrointestinal bleeding, or ulcers (20). However, further studies are needed to investigate the mechanism of action for SMS in the treatment of gout. We have recently shown that resident macrophages trigger the inflammation in gouty arthritis (21) while depletion of tissue resident macrophages or blocking M1 macrophages polarization significantly decreased IL-1β expression and neutrophil infiltration (22). In this study, we aim to examine anti-inflmmatory effects of SMS in gouty arthritis and further investigate the potential mechanism of SMS on macrophages polarization.

## Material And Methods

### SMS Preparation

Simiao San were purchased from Jiling Zixin Pharmaceutical Industrial Company, LTD. (Jiling,China) and authenticated by Guang’anmen Hospital, China Academy of Chinese Medical Sciences (Beijing, China). SMS comprises a mixture of 66.7 g of *Phellodendron chinese* SCHNEID, 33.3 g of *Atractylodes lancea*, 33.3 g of *Coix lacryma-jobi* L., and 66.7 g of *Achyranthes bidentata* BL. SMS medicinal juice was prepared using a well established protocol (18). The four herbs were mixed with distilled water for 2 h, added to a volatile oil extractor, refluxed, and extracted twice for 1.5 h each. A 7-fold excess of distilled water was added for the first extraction, whereas a 6-fold excess of distilled water was added for the second extraction. The mixture was then filtered, sealed for storage, concentrated, and mixed with the volatile oil. Finally, we dispensed the mixture into a 1 g/mL medicinal juice and sterilized it for packaging.

### Mass Spectrometry

Mass spectrometry as performed using an Agilent 1290 UHPLC and 6530 QTOF-MS/MS LC-MS system (Agilent Technologies, Santa Clara, CA, USA), and an Agilent Eclipse Plus C18 column (2.1 × 100 mm, 1.8 μm) column (Agilent Technologies) was used for the acquisition of metabolic data. Gradient elution was performed using 0.1% formic acid and methanol.

For sample preparation, 1 mL of SMS concentrate was added to 4 mL of methanol solution, and the mixture was sonicated for 30 min, centrifuged, and filtered through a 0.1-µm microporous filter.

Liquid chromatography–mass spectrometry was performed on an Agilent 1290 UHPLC and 6530 QTOF-MS/MS system with a 2.1 m × 100 mm × 1.8 μm Agilent Eclipse Plus C18 column. The atomizing temperature was 350°C, the nebulizer flow rate was 10 L/min, the atomization gas pressure was 30 psi, the capillary voltage was 3500 V, the Skimmer voltage was 65 V, the octopole RF voltage was 750 V, and the papillary outlet voltage was 150 V. The data matrix comprised the retention times, m/z 50–1500 values, and the corresponding peak areas for subsequent statistical analysis.

### MSU Crystal Preparation

MSU crystals were prepared as we previously reported (23) and then assessed using compensated polarized light microscopy. Endotoxin was present at <0.015 EU/mL in the MSU crystal preparations, as determined using the Limulus amebocyte lysate assay (Sigma-Aldrich, St Louis, MO, USA). Before each experiment, the MSU crystals were milled and then sterilized for 2 h.

### Cell Culture

THP-1 cells were purchased from the Cell Bank of the Chinese Academy of Sciences (Shanghai, China) and cultured in complete medium comprising RPMI supplemented with 2 mM l-glutamine, 100 units/mL penicillin, 100 μg/mL streptomycin, and 10% fetal bovine serum (FBS) (Gibco BRL, Grand Island, NY, USA). THP-1 cells were used after treatment with 100 ng/μL PMA for 48 h. Cells were seeded in 24-well culture plates (2×10^5^ cells/mL/well), pretreated with 1 or 2 μg/mL SMS for 12 h, and then incubated with 100 μg/mL MSU for 48 h. To block the PI3K/Akt pathway, cells were pretreated with 50 μM LY294002 (Selleckchem) for 1 h before MSU treatment. SMS was added for 30min after LY294002 treatment.

### Animals

Specific pathogen-free male C57BL/6 mice (6-8 weeks old) were purchased from the Shanghai Laboratory Animal Center (Chinese Academy of Sciences, China) and maintained at the Animal Center of Shanghai Medical School of Fudan University. This study was approved by the Ethics Committee of the Department of Laboratory Animal Science, Fudan University (No. 20160981A302).

To establish mice with gout, 50 μL of an MSU suspension (1 mg/50 μL Normal saline) was intra-articularly injected in the right footpad of each animal, whereas the left footpad was injected with 50 μL of Normal saline (N.S). The joint index evaluation was performed as we previously reported (23–27). In the SMS treatment group, mice were intra-gastrically injected with a low dose (1 mg/kg·day) or high dose (10 mg/kg·day) of SMS for 14 days before MSU administration, samples were collected at 8h after MSU injection. The foot joint tissues were immediately isolated, snap-frozen in liquid nitrogen, and stored at -80°C.

Hyperuricemic mice were established *via* treatment with yeast polysaccharide (YP) and potassium oxonate (OP) for 3 weeks as previously described (28). Mice were orally feed containing 1/4 yeast polysaccharide (Sigma, USA) or intraperitoneally injected with potassium oxonate (250 mg/kg; OP, Sigma, USA) at 8:00 a.m. every day. In the SMS treatment group, different concentrations (1 or 10 mg/kg·day) of SMS were intra-gastrically administered to mice simultaneously with OP for 3 weeks. Samples were collected after the last drug administration. Blood samples were obtained from the eye socket vein of each mouse and centrifuged at 2500rpm for 10 min at 4°C. The kidney tissues were immediately isolated, snap-frozen in liquid nitrogen, and stored at -80°C.

### Histological Studies and Immunostaining

After the joint index evaluation, mice were sacrificed, and joint tissue and kidneys sections were prepared for hematoxylin and eosin (H&E) staining and immunostaining. The paraffin-embedded sections were cut at a thickness of 4 μm and placed on positively charged slides for staining. In H&E staining, inflammatory cells were counted using ImageJ software (version 1.51p, National Institutes of Health, Bethesda, MD, USA).

For immunofluorescence, slides were placed in target retrieval solution, and staining was performed manually at room temperature with anti–Arg-1 antibody (Clone #CI:A3-1, 1:500 dilution, Abcam, Cambridge, MA, USA) or anti-F4/80 antibody (Clone #SP156, 1:400 dilution, Abcam, Cambridge, MA, USA). Arg-1 expression was visualized using Alexa Fluor 647 secondary antibody (ab150115, 1:400 dilution, Abcam, Cambridge, MA, USA). F4/80 expression was visualized using Alexa Fluor 488 secondary antibody (ab150077, 1:400 dilution, Abcam, Cambridge, MA, USA). Slides were counterstained with Vectashield hard-set mounting medium with DAPI (Vector Laboratories, Burlingame, CA, USA) and kept in the dark at 4°C until visualization using fluorescence microscopy. Images were obtained *via* optical sectioning using a Nikon epifluorescence microscope for analysis. F4/80^+^/Arg-1^+^ cells (M2 macrophage) analysis was performed using ImageJ software from randomly chosen fields [the average of 5 to 10 fields (× 200) in the section]. F4/80^+^/Arg-1^+^ cells were determined by using a standardized custom histogram-based colored thresholding technique and then subjected to “particle analysis” using ImageJ.

For immunohistochemistry, slides were placed in target retrieval solution, and staining was performed manually at 4°C for 18 hours with Akt (Clone #11E7, 1:1000 dilution, Cell Signaling Technology, Boston, USA). Then goat anti-Rabbit IgG H&L (HRP) (ab6721, 1:500 dilution, Abcam, Cambridge, MA, USA) was added and incubation continued for 20 minutes. Staining was visualized using DAB and the sections were then observed. Finally, sections were further dyed and sealed with hematoxylin. Images were obtained *via* optical sectioning using a Nikon epifluorescence microscope for analysis. To display the results more intuitively, the average optical density of positive areas (40×,100X) was calculated using ImageJ software.

### Determination of Serum Uric Acid (SUA) and Creatinine Levels

SUA concentrations and creatinine levels were separately determined using colorimetric method (#MAK077, #MAK080, Sigma, USA).

### Real-Time Quantitative PCR (qPCR) Detection System

Total RNA was extracted from joint tissue or cells with Trizol (Invitrogen, Carlsbad, CA, USA) according to the manufacturer’s instructions, and reverse translation was conducted using an iScript™ cDNA Synthesis Kit (Bio-Rad, Hercules, CA, USA). The PCR primers (BioTNT, Shanghai, China) used for qPCR were as shows in **Supplementary Table 1**.

The RNA expression in samples was normalized to that of control housekeeping genes (murine Gapdh), and the relative mRNA levels of target genes were calculated using the 2^−ΔΔCt^ method.

### Western Blot Analysis

The joint samples were homogenized by tissue tearor, and added with 10 equivalent volumes of RIPA lysis buffer supplemented with 1 mM PMSF (protease inhibitor) in an ice bath for 30 min and then centrifuged (12,000*g*, 10 min) to extract total proteins. The protein concentration was determined using a BCA protein assay kit (Beyotime Biotechnology, Shanghai, China). Mouse joint tissues or cell lysates from different groups were prepared, and equal aliquots of protein extract were electrophoresed *via* SDS-PAGE. Protein levels were expressed as a ratio of the protein level to that of β-actin.

The following antibodies were obtained from commercial sources as indicated: NLRP3(Clone #Ala306, 1:1000 dilution, Cell Signaling Technology, Boston, USA), p-Akt (Ser473) (Clone #587F11, 1:1000 dilution, Cell Signaling Technology, Boston, USA), Akt (Clone #11E7, 1:1000 dilution, Cell Signaling Technology, Boston, USA), β-actin(Clone #8H10D10, 1:1000 dilution, Cell Signaling Technology, Boston, USA), anti-rabbit IgG HRP-conjugated antibody(#7074, 1:5000 dilution, Cell Signaling Technology, Boston, USA), anti-moude IgG HRP-conjugated antibody(#7076, 1:5000 dilution, Cell Signaling Technology, Boston, USA). The immunoblots were visualized *via* ECL (Pierce, Rockford, IL, USA). The results were normalized to β-actin. Gel quantification was performed using the ImageJ software.

### Statistical Analysis

Data were analyzed using GraphPad Prism 6.0 (GraphPad Software, La Jolla, CA, USA) and presented as the mean ± SEM or median (range). Repeated-measures analysis of variance followed by the Student-Newman-Keuls test was used for *post hoc* analyses of differences among groups. P < 0.05 indicated statistical significance.

## Results

### SMS Ameliorate MSU-Induced Acute Gouty Arthritis in Mice

**Figure 1:**
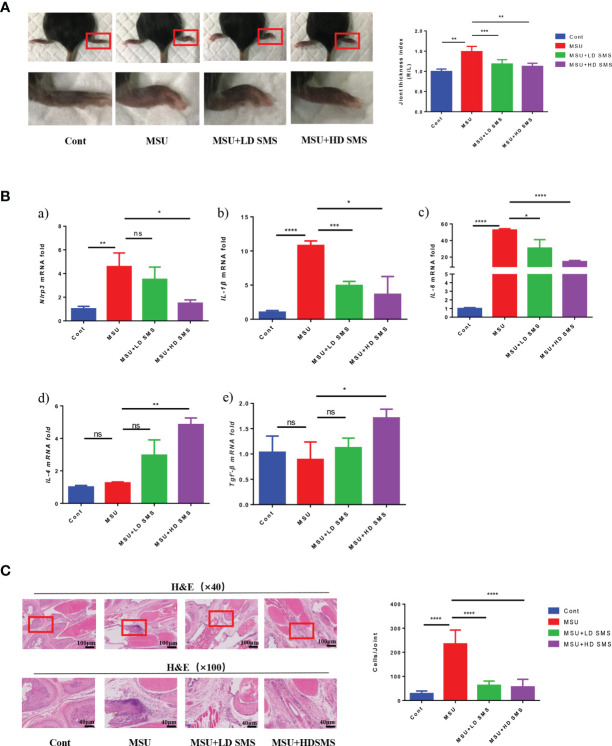
SMS ameliorate MSU induced acute gouty arthritis in mice. Joint swelling and the joint thickness index at 8 hours after MSU injection in the mice **(A)**. The mRNA expression of *Nlrp3*, *IL-1β*, *IL-6*, *IL-4*, *Tgf-β* in the injured joints of the mice **(B)**. H&E staining, and inflammatory cells infiltration in the joints **(C)**. (mean ± SEM. ns p > 0.05, *p < 0.05, **p < 0.01, ***p < 0.001, ****p < 0.0001; n = 6/group; LD SMS: 1mg/kg.day low dose SMS; HD SMS:10mg/kg.day high dose SMS).

Mice were pretreated with different doses of SMS (1 or 10 mg/kg.day) for 2 weeks, and then acute gouty arthritis was induced by MSU. Eight hours after MSU injection, joint thickness and the mRNA expression of *Nlrp3*, *IL-6*, *IL-1β*, *IL-4*, *Tgf-β* in the joints were significantly elevated. The joint swelling was significantly reduced in the mice treated with SMS compared with that in the MSU mice (**Figure 1A**). Moreover, the over-expression of *Nlrp3*, *IL-6*, and *IL-1β* mRNA in MSU-injured joints was inhibited by SMS, especially the high-dose SMS (10mg/kg.day). Notably, the anti-inflammatory factors, IL-4 and Tgf-β, were further increased by SMS treatment (**Figure 1B**). H&E pathological staining further revealed that inflammatory cell infiltration in gouty joints was notably inhibited by SMS pretreatment, especially in the SMS high-dose group (**Figure 1C**).

### SMS Ameliorate Gouty Arthritis *via* the PI3K/Akt Pathway

**Figure 2:**
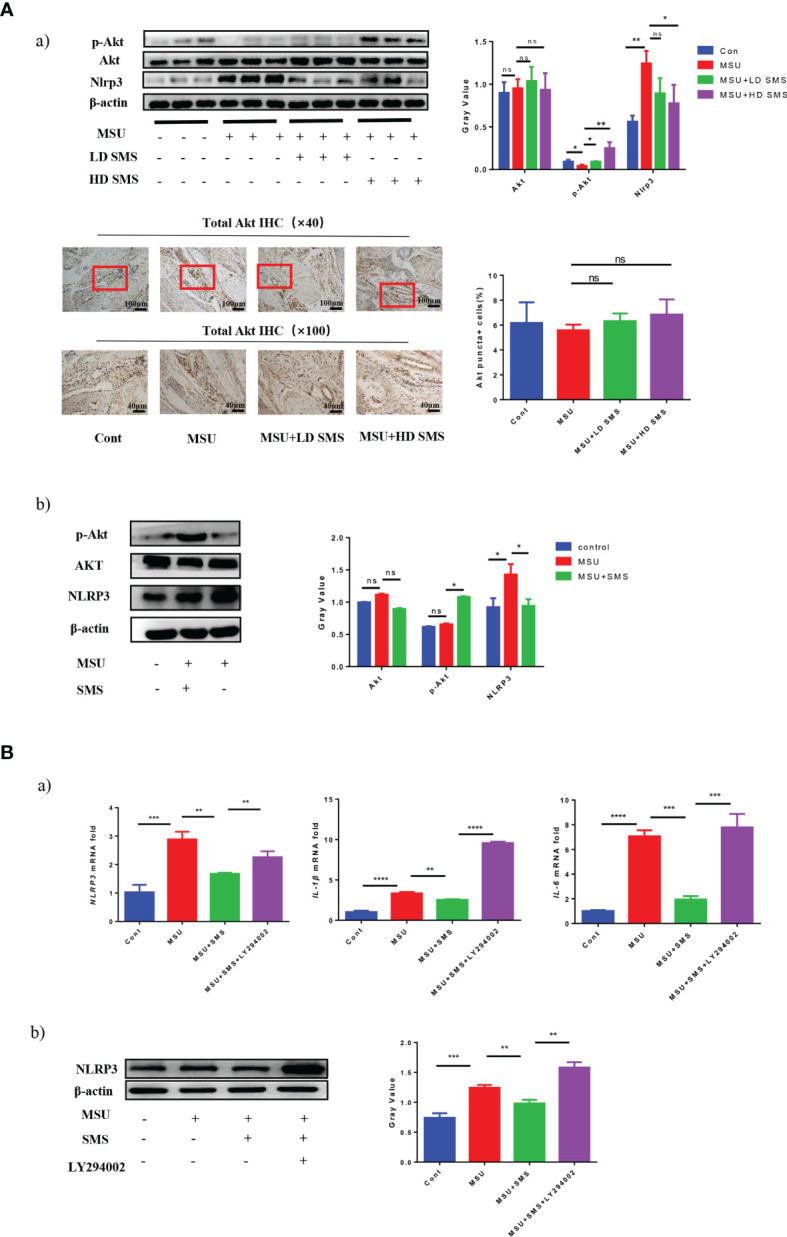
SMS ameliorate gouty arthritis by PI3K/Akt pathway. The expression of Akt, p-Akt (Ser473) and Nlrp3 protein was detected by Western blot analysis in the MSU injured joints of the mice, and total Akt expression was detected by IHC staining **(Aa)**. *In vitro*, p-AKT (Ser473), AKT and NLRP3 protein was detected by Western blot analysis in the THP-1 cells treated by MSU with/without SMS **(Ab)**. Akt activation was inhibited by LY294002, the expression of NLRP3, IL-6 and IL-1β were detected by RT-PCR **(Ba)** or Western blot analysis **(Bb)**. (mean ± SEM. ns p > 0.05, *p < 0.05, **p < 0.01, ***p < 0.001, ****p < 0.0001; n = 6/group; LD SMS: 1mg/kg.day low dose SMS; HD SMS:10mg/kg.day high dose SMS).

Nlrp3 protein expression was significantly increased in the injured joints of MSU mice, whereas p-Akt (S473) expression was decreased. SMS treatment suppressed the upregulation of Nlrp3, but further increased the expression of p-Akt (S473), especially in the SMS high-dose group (10mg/kg) (**Figure 2** **Aa**). No significant difference of the total Akt expression was detected in mice joint of all the groups.

*In vitro*, inflammatory factors NLRP3, IL-6, and IL-1β were also reduced by SMS treatment in the MSU stimulated THP-1 cells, while p-AKT (S473) was upregulated (**Figures 2** **Ab**, **2Ba**). However, the inhibitory effects of SMS on NLRP3 were notably suppressed by pre-treatment with the PI3K/Akt inhibitor LY294002 (**Figure 2** **Bb**).

### SMS Promote M2 Macrophage Polarization

**Figure 3:**
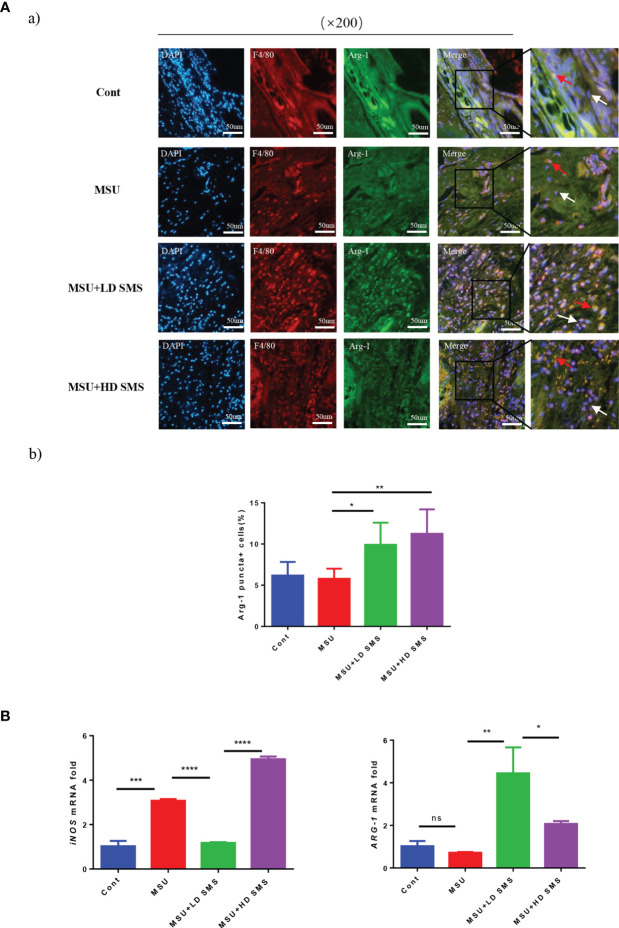
SMS promote M2 macrophage polarization. IF co-localization staining of F4/80, Arg-1, DAPI in joint sections of mice were performed to estimate macrophage polarization **(Aa)**, the red arrows represent F4/80^+^/Arg-1^+^ cells, white arrows represent F4/80^+^/Arg-1^-^ cells. The F4/80^+^/Arg-1^+^ cells were counted **(Ab)**. The mRNA expression of ARG-1 and iNOS were detected by RT-PCR **(B)**. (mean ± SEM. ns p > 0.05, *p < 0.05, **p < 0.01, ***p < 0.001, ****p < 0.0001; n = 6/group; LD SMS: 1mg/kg.day low dose SMS; HD SMS:10mg/kg.day high dose SMS).

To identify the macrophage polarization, joint tissue from mice was subjected to double immunofluorescence staining for the marker F4/80 and Arg-1. F4/80^+^/Arg-1^+^ cells were ubiquitously observed in mouse joints (**Figure 3** **Aa**), and were significantly increased by SMS treatment (**Figure 3** **Ab**). In MSU-treated THP-1 cells, iNOS expression was induced, whereas ARG-1 remained stable. Upon SMS administration, iNOS expression was inhibited but ARG-1 was elevated. After blocking Akt activation with LY294002, Arg-1 upregulation and the anti-inflammatory effects of SMS were simultaneously suppressed in macrophages (**Figure 3B**).

### SMS Inhibit Hyperuricemia in Mice

**Figure 4:**
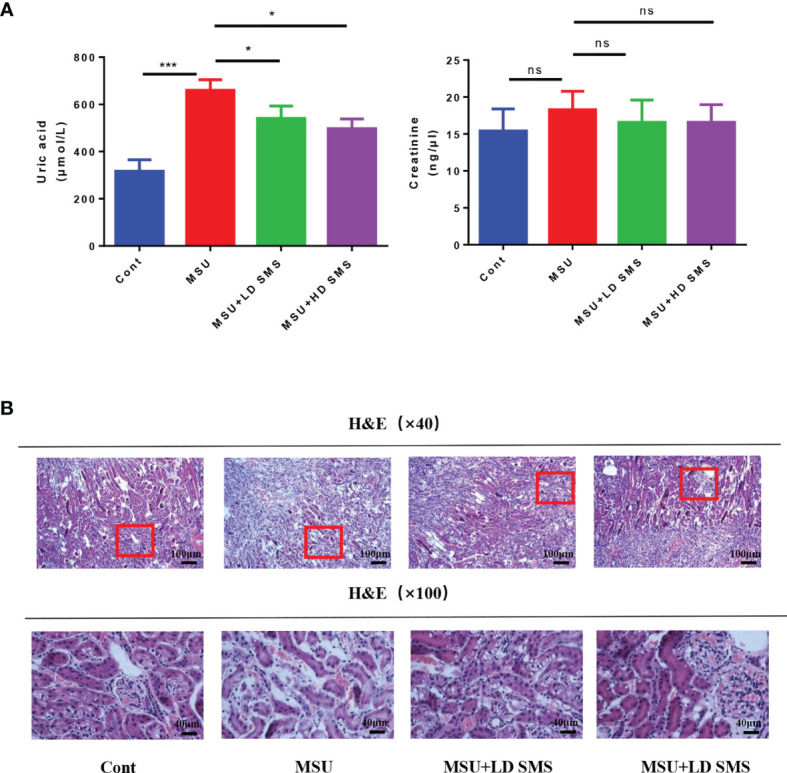
SMS inhibit hyperuricemia in mice. Serum uric acid and serum creatinine were detected in the different groups of mice **(A)**. H&E staining of kidney in the different groups of mice **(B)**. (mean ± SEM. ns p > 0.05, *p < 0.05, ***p < 0.001; n = 6/group; LD SMS: 1mg/kg.day low dose SMS; HD SMS:10mg/kg.day high dose SMS).

In this study, we further evaluated the effects of SMS on SUA levels. As presented in **Figure 4A**, hyperuricemia was induced in mice treated with PO and YP for 3 weeks. However, hyperuricemia was inhibited when the mice were simultaneously treated with SMS. Compared with the control group, the hyperuricemia and the SMS-treated mice did not show histological changes in the kidney (**Figure 4B**).

### Ecdysone and Estrone Detected in SMS *via* Mass Spectrometry

**Figure 5:**
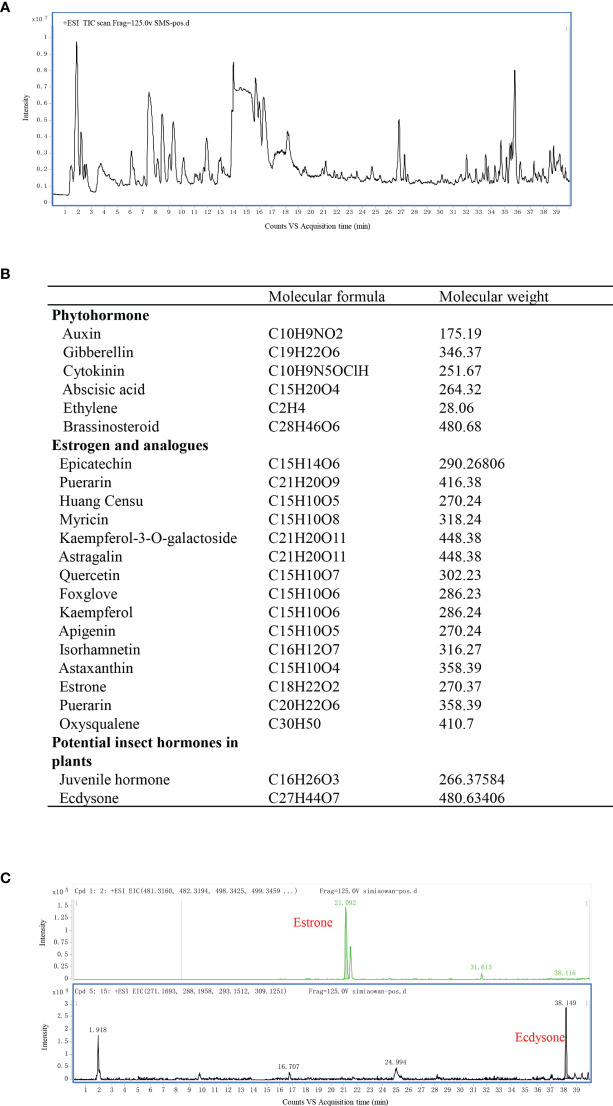
Ecdysone and estrone detected in SMS *via* mass spectrometry. Total Ion Chromatography of SMS was detected by mass spectrometry **(A)**. Common phytohormone, estrogen and its analogs, and other potential hormones in plants to be screened in our study are listed **(B)**. Ecdysone and estrone were detected in SMS **(C)**.

Because of the anti-inflammatory and uric acid lowering effects of SMS, mass spectrometry was used to screen the ingredients responsible for the observed benefits. Total Ion Chromatography of SMS was presented in **Figure 5A** with mass spectrometry. SMS did not exhibit any ion currents of the common ingredients of steroid (the spectra of the ingredients screened were listed in the **Supplement Table 2**) or non-steroidal anti-inflammatory drugs (the spectra of the ingredients screened were listed in the **Supplement Table 3**). Then phytohormones, estrogen and its analogs, and other potential hormones in plants were further examined by mass spectrometry (**Figure 5B**), ecdysone and estrone were finally detected in SMS (**Figure 5C**).

## Discussion

Gout was first recorded in “Gezhiyulun” by DanXi Zhu in 1347 (Yuan Dynasty), and some effective traditional Chinese medicines against gout were described in ancient medical records. Colchicine, NSAIDs, or glucocorticoids were suggested as appropriate first-line therapy for gout flares in the recent recommendations for gout management (8). But the use of these drugs are often limited in gouty patients with concomitant complications such as chronic renal insufficiency, heart problems, gastrointestinal bleeding, or ulcer (29–31). Among the various Chinese medicines against gout, SMS was clinically confirmed to be effective and safe as an anti-inflammatory therapy for gout, and remains in use to the present day (18). In this study, we further researched its effects on gouty mice and examined the potential mechanism of SMS.

Gout is an inflammatory disease caused by serum uric acid oversaturation and MSU crystal irritation (3). Inflammatory cells infiltrated the joints release inflammatory mediators such as cytokines and chemokines, which result in acute gouty inflammation (32–36). Therefore, anti-inflammatory therapy is the primary approach for preventing gouty arthritis. In this study, SMS was found to ameliorate joint swelling and local inflammatory cell infiltration. Expression of the specific inflammatory factors Nlrp3 and IL-1β in gouty joints was notably inhibited by SMS treatment. These results confirmed that SMS is effective against MSU-induced gouty arthritis. Based on its characteristics, SMS may be an effective treatment of gout.

According to our previous research, Akt phosphorylation was increased in the MSU-induced inflammatory environment (21). Studies suggested that activation of the PI3K/Akt pathway inhibits inflammation, and these effects are mainly attributed to its inhibitory effects on nuclear factor-κB activity (37). The results suggested that PI3K/Akt pathway activation may be a beneficial response to inflammation or injury. In our study, Akt phosphorylation at Ser473 was significantly increased after SMS treatment in the injured joints of gouty mice. When PI3K/Akt activation was blocked by the specific inhibitor LY294002 in macrophages, the inhibitory action of SMS on inflammation was consequently suppressed. The results suggested that SMS can prevent gouty inflammation through activating the PI3K/Akt pathway.

Recent studies have shown the involvement of the neutrophils, macrophages and other immune cells in the inflammation progress and alleviation of gout (3). Macrophages usually exhibit distinct phenotypes in different tissue microenvironments (22, 38–40). In previous research, we found that the activation status of macrophages determines their function in gouty inflammation progression or restoration (21). Generally, there are two major phenotypes of activated macrophages depending on the inflammatory microenvironment, classically activated (M1) and alternatively activated (M2) macrophages (41). M1 macrophages exhibit high iNOS expression and mainly play pro-inflammatory roles, whereas M2 macrophages exhibit high Arg-1 expression and play anti-inflammatory roles (42–44). In our research, macrophages exhibited abundant iNOS expression upon MSU stimulation. After SMS administration, MSU-induced iNOS overexpression was suppressed, while Arg-1 was upregulated. It showed that SMS induced macrophage polarization toward M2 phenotype. In macrophages treated with LY294002 to block the PI3K/Akt pathway, Arg-1 expression was consequently inhibited. It suggested that SMS induces M2 macrophage polarization by activating the PI3K/Akt pathway, which might be the mechanism of its anti-inflammatory effects on gout.

From the aforementioned results, SMS was confirmed to inhibit gouty inflammation, which prompted us to examine its potential anti-inflammatory ingredients. However, no such compound was detected in SMS by screening for the common ingredients of steroid and non-steroidal anti-inflammatory drugs *via* mass spectrometry. Then, the spectra of phytohormones, estrogen and its analogs, and potential insect hormones in plants were further screened. Interestingly, ecdysone and estrone were finally detected in SMS.

Ecdysone (20-hydroxyecdysone) is a phytoecdysteroid with biological activity that has not been thoroughly investigated to date. It has been reported to regulate the immune response and inhibit bacterial infection in Drosophila embryos (45, 46). Ecdysone inhibited the inflammatory cascade and oxidative stress process in rats with collagen-induced rheumatoid arthritis (47). Based on its effect, ecdysone might be the ingredient responsible for the anti-inflammatory effects of SMS against gout.

In this study, estrone was another plant hormone detected in SMS. It is a relatively abundant hormone that is widely distributed in tissues of animal and plant origin (48). As the predominant estrogen, estrone was reported to regulate metabolism (49) and increase uric acid excretion (50). Women of productive age are well known to seldom experience hyperuricemia or gout because of the regulatory effects of estrogen on SUA content (51). SMS might prevent hyperuricemia because of being rich in estrone. In this study, we found that SMS treatment in hyperuricemia mice resulted in significantly decreases in SUA levels. This result was consistent with previous studies in which SMS ameliorated high fructose-induced insulin resistance, dyslipidemia, high uric acid levels, and kidney injury in rats (52). However, further research is needed to verify the specific role of ecdysone and estrone in gout and hyperuricemia.

## Conclusion

This study demonstrated that SMS ameliorate MSU-induced gouty arthritis and inhibit hyperuricemia. The anti-inflammatory mechanism of SMS might involve M2 macrophages polarization *via* activation of the PI3K/Akt pathway. These findings provided insight for developing therapeutic approaches to treat gout. The plant hormones ecdysone and estrone were detected in SMS. However, further research is needed to clarify their anti-inflammatory function and mechanism of action for uric acid lowering effects.

## Data Availability Statement

The datasets presented in this study can be found in online repositories. The names of the repository/repositories and accession number(s) can be found in the article/**Supplementary Material**.

## Ethics Statement

The animal study was reviewed and approved by The Ethics Committee of the Department of Laboratory Animal Science, Fudan University (No. 20160981A302).

## Author Contributions

LC and TZ performed mouse experiments and *in vitro* experiments. YXu, LX, YC, and FQ contributed to the experimental design. YXi performed mass spectrometry. WW conducted the statistical analysis and computational data analysis. MH and QJ contributed to the data interpretation. LL contributed to the revise the manuscript with significant input. XZ and HZ contributed to manuscript drafting and conceived the study. All authors reviewed the manuscript. All authors read and approved the final manuscript.

## Funding

This work was supported by the National Natural Science Foundation of China (82071830) and Research Funding from the Shanghai Hospital Development Center (SHDC12016227) and Shanghai Municipal Health Commission (20204Y0428) and Young Elite Scientists Sponsorship Program by CACM(CACM-2020-QNRC2-05).

## Conflict of Interest

The authors declare that the research was conducted in the absence of any commercial or financial relationships that could be construed as a potential conflict of interest.

## Publisher’s Note

All claims expressed in this article are solely those of the authors and do not necessarily represent those of their affiliated organizations, or those of the publisher, the editors and the reviewers. Any product that may be evaluated in this article, or claim that may be made by its manufacturer, is not guaranteed or endorsed by the publisher.
