# Supplementary material for: The Anti-Inflammatory and Uric Acid Lowering Effects of Si-Miao-San on Gout
Source: Front Immunol. 2022 Jan 5;12:777522. doi: 10.3389/fimmu.2021.777522 (PMC8769502; doi:10.3389/fimmu.2021.777522)
Supplement: Supplementary file 1 [file DataSheet_1.docx]

**Supplementary Table 1: Real-time PCR primers in this study**

| PCR primers | Forward (5′-3′) | Reverse (5′-3′) |
| --- | --- | --- |
| Mouse *IL-1b* | GTACAAGGAGAACCAAGCAA | CCGTCTTTCATTACACAGGA |
| Mouse *Nlrp3* | ATTACCCGCCCGAGAAAGG | CATGAGTGTGGCTAGATCCAAG′ |
| Mouse *IL-6* | GGCGGATCGGATGTTGTGAT- | GGACCCCAGACAATCGGTTG |
| Mouse *IL-4* | GGTCTCAACCCCCAGCTAGT | GCCGATGATCTCTCTCAAGTGAT |
| Mouse  *Tgf-β* | ATGGCGCAAAACAGTCCACA | TGTAACATGCACTGGGATACCA |
| Mouse *Gapdh* | GGAGCGAGATCCCTCCAAAAT | GGCTGTTGTCATACTTCTCATGG |
| Human *IL-1β* | ATGATGGCTTATTACAGTGGCAA | GTCGGAGATTCGTAGCTGGA |
| Human *IL-6* | ACTCACCTCTTCAGAACGAATTG | CCATCTTTGGAAGGTTCAGGTTG |
| Human *NLRP3* | ATTACCCGCCCGAGAAAGG | CATGAGTGTGGCTAGATCCAAG |
| Human *ARG1* | CAGATATGCAGGGAGTCACC | CAGAAGAATGGAAGAGTCAG |
| Human *iNOS* | AGCTGAACTTGAGCGAGGAG | GGAAAAGACTGCACCGAAGA |
| Human *GAPDH* | AACTCCCACTCTTCCACCTTCG | TCCACCACCCTGTTGCTGTAG |

**Supplementary Table 2: The spectra of common steroid screened in SMS with mass spectrometry analysis**

| Medicine | Molecular formula | Molecular weight |
| --- | --- | --- |
| Prednisolone/Cortisone | C21H28O5 | 360.2009 |
| Flumamide | C17H16F3NO2 | 376.2120 |
| Betamethasone / Dexamethasone | C22H29FO5 | 392.2071 |
| Cortisone acetate / Prednisolone acetate | C23H30O6 | 402.2115 |
| Flumic acid | C16H13ClFNO5 | 410.1977 |
| Methylprednisolone acetate | C24H32O6 | 416.2271 |
| Budesonide | C25H34O6 | 430.2428 |
| Triamcinolone acetonide / Betamethasone acetate / Dexamethasone acetate | C24H31FO6 | 434.2176 |
| Triamcinolone acetonide / Betamethasone valerate | C27H37FO6 | 476.2642 |
| Fluoroacetic acid acetate / Difluranone diacetate | C2ClF2O2 | 494.2187 |
| Mometadonate/Beclomethasone dipropionate | C28H37ClO7 | 520.2298 |
| Hydrocortisone | C23H32O6 | 362.2166 |
| Prednisone | C21H26O5 | 358.1853 |
| Methylprednisolone | C22H30O5 | 374.2166 |
| Triamcinolone | C21H27FO6 | 394.1864 |
| Fluticasone propionate | C25H31F3O5S | 500.1917 |
| Flumic acid | C16H13ClFNO5 | 410.1733 |
| Beclomethasone | C22H24D5ClO5 | 408.1576 |
| Hasinide | C42H58Cl6Nb2O4 | 454.1995 |
| Divcot | C25H31NO6 | 441.2224 |
| Beclomethasone | C22H24D5ClO | 408.1776 |

**Supplementary Table 3: The spectra of common NSAIDs screened in SMS with mass spectrometry analysis**

| Medicine | Molecular formula | Molecular weight |
| --- | --- | --- |
| Aspirin | C9H8O4 | 181.0495 |
| Phenylzoline | C19H20N2O2 | 309.1598 |
| Hydroxy phenylbutazone | C19H20N2O3 | 325.1547 |
| Ibuprofen | C13H18O2 | 207.1380 |
| Naproxen | C14H14O3 | 231.1016 |
| Diclofenac | C14H9Cl2NO | 278.0134 |
| Indomethacin | C19H16ClNO4 | 358.0841 |
| Asimisin | C21H18ClNO6 | 416.0895 |
| Flufenamic acid | C14H10F3NO2 | 282.0736 |
| Mefenamic acid | C15H15NO2 | 242.1176 |
| Meloxicam | C14H13N3O4S2 | 352.0420 |
| Nimesulide | C13H12N2O5S | 309.0540 |
| CGP28238 | C16H13F2NO4S | 354.0606 |
| NS-398 | C13H18N2O5S | 315.1009 |
| L-745337 | C16H13F2NO3S2 | 370.0378 |
| Celecoxib | C17H14F3N3O2S | 382.0832 |
| Rofecoxib | C17H14O4S | 315.0686 |
| Etoricoxib | C18H15ClN2O2S | 359.0616 |
| Valdecoxib | C16H14N2O3S | 315.0798 |
| SC58125 | C17H12F4N2O2S | 385.0628 |
| DUP697 | C17H12BrFO2S2 | 410.9519 |
| RWJ63556 | C11H10FNO3S2 | 288.0159 |
